# Molecular Pathways Modulated by Mesenchymal Stromal Cells and Their Extracellular Vesicles in Experimental Models of Liver Fibrosis

**DOI:** 10.3389/fcell.2020.594794

**Published:** 2020-12-08

**Authors:** Giulia Chiabotto, Chiara Pasquino, Giovanni Camussi, Stefania Bruno

**Affiliations:** ^1^Department of Medical Sciences, University of Turin, Turin, Italy; ^2^Molecular Biotechnology Center, University of Turin, Turin, Italy

**Keywords:** mesenchymal stem cell, collagen, α-SMA, hepatic stellate cell, fibrosis, inflammation, microvesicles, exosomes

## Abstract

End-stage liver fibrosis is common to all chronic liver diseases. Since liver transplantation has several limitations, including lack of donors, immunological rejection, and high medical costs, therapeutic alternatives are needed. The administration of mesenchymal stromal cells (MSCs) has been proven effective in tissue regeneration after damage. However, the risk of uncontrolled side effects, such as cellular rejection and tumorigenesis, should be taken into consideration. A safer alternative to MSC transplantation is represented by the MSC secretome, which retains the same beneficial effect of the cell of origin, without showing any considerable side effect. The paracrine effect of MSCs is mainly carried out by secreted particles in the nanometer range, known as extracellular vesicles (EVs) that play a fundamental role in intercellular communication. In this review, we discuss the current literature on MSCs and MSC-EVs, focusing on their potential therapeutic action in liver fibrosis and on their molecular content (proteins and RNA), which contributes in reverting fibrosis and prompting tissue regeneration.

## Introduction

Chronic liver disease (CLD) may be caused by different types of injury (viral infection, alcohol abuse, NASH, ischemic injury, chemical compounds, autoimmune and genetic diseases, etc.), and it is one of the major global health problems that causes about 2 million deaths per year worldwide (Asrani et al., 2019; Roehlen et al., 2020). Fibrosis is the main characteristic of CLD and is due to an excessive ECM accumulation, which compromises the normal morphology and function of the liver. Excessive ECM deposition is due to a persistent activation of myofibroblasts that proliferate and produce different matrix components (type I and III collagen, fibronectin, laminin, and proteoglycans) (Roehlen et al., 2020).

Hepatocytes are proliferative cells that support the physiological renewal of liver parenchyma. In case of injury, proliferation is due to the activation of resident HpSCs, which are quiescent during physiological turnover of the organ (Ibrahim et al., 2018). Different studies have observed only a marginal contribution of these resident progenitor cells in ameliorating the damage in several models of hepatocellular injury. In particular, the HpSCs are not able to totally regenerate the hepatocytes damaged in CLD, especially in the presence of a massive destruction of the liver tissue (Español-Suñer et al., 2012). For this reason, an exogenous treatment that supports the regeneration of the altered tissue is demanded.

A possible therapeutic option is the use of MSCs that can be isolated from various adult tissues, such as bone marrow (BM), adipose tissue, muscle, periosteum, umbilical cord, blood, and liver (Puglisi et al., 2011). MSCs were originally described as a rare population of cells in the BM with fibroblast-like morphology (Friedenstein et al., 1974) and a characteristic pattern of cell-surface antigens (Pittenger et al., 1999). Besides their self-replicating ability, MSCs can differentiate in mesenchymal (osteoblasts, chondrocytes, adipocytes, and myocytes) and non-mesenchymal cells (cardiac, neural, renal, and hepatic cells) (Krause, 2002; Alhadlaq and Mao, 2004). These peculiar properties combined with the immunoregulatory activity of MSCs make these cells ideal candidates for regenerative therapy of various diseases, including liver fibrosis (Eom et al., 2015; Han et al., 2019).

In this review, we summarize recent findings in the field of MSCs application as anti-fibrotic strategy and on molecular pathways modulated by MSCs and their secretome, in particular by EVs.

## Cells and Molecular Pathways Involved in Hepatic Fibrosis

Different types of hepatic cells contribute to fibrosis development. Hepatocyte death is the initial event in liver diseases that conducts to damage progression. Dead hepatocytes release different molecules (intracellular proteins, ATP, nucleic acids, mitochondrial or nucleic compounds) collectively named DAMPs (Mihm, 2018). These intracellular compounds negatively influence the neighboring cells, such as HSCs and Kupffer cells, favoring cell activation and fibrosis development. Moreover, a pro-fibrogenic signal can be triggered by apoptosis of hepatocytes by the activation of Fas death receptor (Feldstein et al., 2003) and by the release of apoptotic bodies. These can be absorbed by Kupffer cells and by HSCs to activate pro-fibrogenic signals (Canbay et al., 2003; Zhan et al., 2006).

Hepatic stellate cells are the key players in hepatic fibrosis development, since they represent the major source of myofibroblast precursors. In physiological conditions, HSCs are perisinusoidal non-proliferating cells characterized by numerous lipid droplets containing vitamin A in the cytoplasm. Different types of injury may induce HSC activation. In this case, HSCs start to proliferate, acquire a contractile myofibroblast phenotype, express α-SMA, and produce ECM components and pro-inflammatory cytokines (Mederacke et al., 2013). Other important sources of myofibroblasts may be portal fibroblasts (Wells et al., 2004), BM-derived cells (Forbes et al., 2004), and cells derived from hepatocytes or cholangiocytes by EMT (Zeisberg et al., 2007). In addition, the production of pro-inflammatory cytokines induces the recruitment and the activation of other important cells in fibrosis development, such as platelets, endothelial cells, and infiltrating immune cells that may amplify the pro-fibrogenic environment and contribute to support HSC and myofibroblast activation state. Hepatic macrophages, which can be liver resident (Kupffer cells) or monocyte-derived (Krenkel and Tacke, 2017), play a central role in this fibrosis development. Macrophages may be classified into pro-inflammatory macrophages (M1) and immunoregulatory macrophages (M2). Hepatic macrophages may shift from M1 to M2 phenotypes in response to different stimuli, and different macrophage subpopulations may coexist and contribute to different phases of fibrosis (Sun et al., 2017).

A complex network of cytokines and pathways is responsible for HSC activation and for induction of fibrogenic alterations. The most important growth factors and pathways involved in fibrogenesis are the PDGF, the TGF-β, the inflammasome NLRP3–caspase-1, and the Wnt/β-catenin signaling pathway (Dewidar et al., 2019; Roehlen et al., 2020). Moreover, resident immune cells produce under stimulation the TNF-α, the interleukins (IL-6 and IL-1α), and the CCL2, that trigger the activation of Kupffer cells and the recruitment of monocyte-derived macrophages from blood (Roehlen et al., 2020). In physiological conditions, PDGF is produced by platelets. Different types of liver injury induce the production of PDGF also by Kupffer cells, endothelial cells, and activated HSCs, and PDGF receptor is expressed by HSCs (Campbell et al., 2005; Hayes et al., 2014). The binding of PDGF to its receptor triggers the activation of several signaling pathways, including the PI3K/Akt, the JAK/STAT, and the Ras/Raf system. These different pathways regulate downstream the expression levels of the key pro-fibrotic genes, such as type I collagen α1 chain, MMPs, tissue inhibitors of metalloproteinases (TIMPs), and also apoptosis regulators (Bcl-2) that favor the survival and proliferation of myofibroblasts (Ying et al., 2017). Endothelial cells, macrophages, and hepatocytes can synthesize TGF-β, as a latent precursor. The inactive form of TGF-β, bound to the latency-associated protein, is stored in the ECM. TGF-β becomes active when cleaved by a specific protease. The active form binds to TGF-β receptor type II, which recruits the TGF-β receptor type I, with downstream activation of SMAD proteins. In particular, the activation of SMAD3 by phosphorylation at its C-terminus is considered the main fibrogenic pathway (Liu et al., 2006; Fabregat et al., 2016). Furthermore, the activation of the SMAD3-dependent TGF-β signaling pathway in hepatocytes contributes to fibrosis development, especially in NASH, by inducing hepatocyte death and lipid accumulation. SMAD6 and SMAD7, which negatively regulate TGF-β signaling, are considered as anti-fibrotic factors (Yang et al., 2014).

Another important pathway involved in hepatic fibrosis development is the Wnt/β-catenin signaling. β-Catenin is an adhesion molecule that can also act as a transcription factor. After activation, Wnt binds the receptor Frizzled and forms a complex, which inhibits β-catenin degradation causing its translocation to the nucleus that activates target genes transcription by recruiting cyclic AMP-response element binding (CREB) protein (Nishikawa et al., 2018). In hepatic damage, the Wnt signaling pathway is activated in the HSCs and may contribute to fibrosis development by upregulating α-SMA and collagen expression (Berg et al., 2010).

Despite the increasing number of studies deeply investigating the molecular mechanisms of liver fibrosis development, an approved drug to counteract liver fibrosis is still missing. Anti-fibrotic therapies have to focus on different mechanisms of action: hepatocyte protection, the inhibition of HSC activation, the consequent deposition of fibrotic molecules, and the modulation of inflammation.

## MSC Treatment Counteracts Liver Fibrosis Development

Several studies demonstrated that the injection of MSCs improved liver fibrosis and enhanced liver functionality by reducing hepatocyte apoptosis, prompting hepatocyte regeneration, and regulating inflammatory responses in different animal models of CLD (Table 1). In particular, numerous published results demonstrated the reduction of TGF-β1 and α-SMA gene expression in liver tissue after treatment with MSCs (Rabani et al., 2010; Jang et al., 2014; Winkler et al., 2014; Idriss et al., 2018; Fathy et al., 2020; Sun et al., 2020).

**TABLE 1:** Anti-fibrotic effect of MSCs from different sources in chronic liver injury.

**Type of cells**	**Source**	**Liver fibrosis model**	**Mechanism of action**	**References**
Murine MSCs	BM mononuclear cell fraction selected by plastic adherence	Intraperitoneal (i.p.) administration of a 1.0 ml/kg dose of CCl4 twice a week for 4 weeks	Reduction of collagen deposition by downregulating α-SMA and TIMP-1 gene expression	Rabani et al., 2010
Murine hepatocyte-differentiated BM-MSCs (in the presence of HGF, FGF4, and EGF)	Tibia and femur of Sprague–Dawley (SD) rats	0.5 ml/kg CCl4 was injected subcutaneously into adult male SD rats (320 ± 20 g) twice a week for 4 weeks	Improvement of liver function by modulating the gene of ECM remodeling (MMP-2, MMP-9, and TIMP-1), reducing the expression of IL-1β, IL-6, TNFα, and TGF-β, and increasing IL-10 and HGF	Zhao et al., 2012
Human BM-MSCs and human MSCs	Purchased from Lonza	CCl4 dissolved in corn oil (1:3) twice a week for 6 weeks for the last 4 weeks	Fibrosis reduction by improvement of MMP-9, which degrades the ECM, and downregulation of αSMA, TNFα, and TGF-β, markers of activated HSCs	Tanimoto et al., 2013
Murine MSCs and hepatocytes	Tibia and femur of 2-month-old C57BL/6 mice	*In vitro*: hepatocytes were seeded on a 6-well collagen-coated plate (1 × 104 cells/cm^2^) and were subjected to injury with 3 mM and 5 mM CCl4. *In vivo*: female C57BL/6 mice (6–8 weeks old) intraperitoneally injected with 1 ml/kg CCl4 in olive oil (1:1) for 4 weeks.	*In vitro*: reduction of apoptotic markers, such as Bax, caspase-3, NF-κB, IL-6, and TNF-α, and increased levels of anti-apoptotic marker Bcl-xl. *In vivo*: increased expression of Bcl-xl and reduction of expression levels of apoptotic markers Bax, caspase-3, NF-κB, and TNF-α	Nasir et al., 2013
Human BM-MSCs	Posterior iliac crest of healthy donors	i.p. injections of TAA (300 mg/kg body weight) twice a week for 12 weeks in SD rats	Recovery from TAA induced fibrosis by decreasing TGF-β1, type I collagen, and α-SMA expression and modulating the TGF-β1/SMAD signaling pathway	Jang et al., 2014
Human hepatocyte-differentiated BM-MSCs	Knee or hip joint of human donors	Immunodeficient male Pfp/Rag2–/– mice that underwent 1/3 partial hepatectomy after 42 days of MCDD	Presence of human hepatocyte-like cells in the mouse liver parenchyma attenuating inflammation markers (TNFα). Reduction of the expression of α-SMA and type I collagen mRNA	Winkler et al., 2014
Human BM-MSCs	BM of human healthy donors	*In vitro* model: co-culture of BM-MSCs with HSCs in transwell condition for 24, 48, and 72 h	Inhibitory effect of BM-MSCs on HSC proliferation and induction of the apoptosis through the inhibition of the TGF-β1/SMAD pathway in HSCs	Zhang et al., 2015
Murine BM-MSCs	Femur of healthy Swiss mice	Male Swiss mice treated by 1.0 ml/kg CCl4 via oral administration 3 times/week (every 2 days) for 11 weeks	Inhibition of fibrogenesis with the reduction of integrin, TGF-β1, and pro-collagen expression	Truong et al., 2016
Adult human BM-MSCs and neonatal human Wharton’s jelly (WJ)-MSCs	BM mononuclear cells from three independent donor aspirations (BM-MSCs) and fresh umbilical cords collected from full-term births (WJ-MSCs)	SD rats i.p. injected with CCl4 at a dose of 2 ml/kg (CCl4:olive oil = 1:1) twice a week for the first 2 weeks, followed by 1 ml/twice a week for the next 6 weeks	Reduction of liver collagen content and improvement of liver architecture, by secreting fibrinolytic metalloproteases, such as MMP-1 and MMP-2	Rengasamy et al., 2017
Rat BM-MSCs	Tibia and fibula of white albino rats	0.2 ml/100 g of CCl4 liquefied in castor oil (40 ml/L) subcutaneously injected twice weekly for 6 weeks in male 6-week-old white albino rats	Recovery of liver function and improved liver fibrosis with prolonged presence of transplanted BM-MSCs in the liver: reduction in the expression of pro-inflammatory cytokines (IL-1β, IL-6, and IFN-γ) and of pro-fibrogenic factors (TGF-β1, α-SMA, and CTGF) and increase in the expression of anti-fibrogenic factors (CK-18 and HGF)	Idriss et al., 2018
Rat BM-MSCs transfected with human MMP-1	BM of SD rats	CCl4 administration in rats at a dose of 1 ml/kg twice/week for 8 weeks	Degradation of hepatic collagen due to significant increased MMP-1 level and suppression of TIMP-1	Du et al., 2018
Rat BM-MSCs	Tibia and femur of rats	Rats i.p. injected with CCl4 (1 ml/kg) dissolved in paraffin oil, twice a week, for 6 weeks (12 doses)	Significant downregulation of Col1a1, AFP, and STAT3 and STAT5 gene, whereas significant improvement of Alb expression	Farouk et al., 2018
Human BM-MSCs cultured under hypoxic (5% O_2_; hypoMSCs) and normoxic (21% O_2_; norMSCs) conditions	Normo: Poietics human MSCs (passage 2) purchased from Lonza. Hypo: StemPro BM-MSCs from Thermo Fisher Scientific	*In vitro*: induced BM-derived co-cultured with MSCs in Transwell 6-well plates for 72 h. *In vivo*: 8-week-old C57BL/6 male mice injected with CCl4 i.p. twice weekly over a 12-week period	Induction of anti-inflammatory markers CD206 and Ym-1 in hypoMSC-treated macrophages. Downregulation of the pro-inflammatory markers TNFα and MCP-1	Kojima et al., 2019
BM-MSCs labeled with super-paramagnetic iron oxide nanoparticles	Femur of male SD albino rats	SD albino rats i.p. injected with CCl4 for 8 weeks	ECM degradation by increased MMP-1 and decreased TIMP-1	Khalifa et al., 2019
Murine BM-MSCs	Tibia and femur of mice C57BL/6	C57BL/6 mice injected with CCl4 (40% in olive oil) at a dose of 1 ml/kg twice/week for 12 weeks	Amelioration of the hypoxic liver microenvironment, improvement of the liver function, and reduction of fibrosis by modulating the TGF-β1/SMADs signaling pathway: reduction of TGF-β1 and SMAD3 expression and increased SMAD7 expression	Zhang et al., 2019
Human ADSCs	Human adipose tissue-derived stromal vascular fraction	Male NOD/SCID mice (NOD.CB17-Prkdcscid/J strain) i.p. injected with 200 mg/kg with TAA 2 times/week for 4 weeks	Induction of liver regeneration and amelioration of fibrosis and inflammation with downregulation of IL-1α, IL-6, and TNF-α and increased expression of HGF and VEGF-A	Choi et al., 2019
Rat BM-MSCs + VEGF	Tibia and femur of 2-week-old SD rats	8-week-old rats i.p. injected with 40% CCl4 at 0.3 ml/100, twice per week, for 12 weeks. VEGF group and the BMSC + VEGF group i.v. injected with VEGF over-expressing adenovirus at 3 × 109 ifu (0.5 ml), once a week for 4 weeks	Low amount of collagen deposition related to low IL-6 mRNA levels; high levels of VEGF and VCAM-1 expression in the hepatic sinusoidal endothelial cells	Yuan et al., 2019
HLSCs	Liver fragment processed in Good Manufacturing Practice (GMP) procedure	NASH induced by MCDD	Significant improvement of liver function and morphology, at histological and molecular levels, by persistence of undifferentiated HLSCs in the liver that induce reduction of α-SMA, type I collagen, and TGF-β expression	Bruno et al., 2019
hDPSCs	Deciduous teeth of healthy pediatric donors	C57BL/6J male, 8-week-old mice i.p. injected with 0.5 mg/kg of CCl4 in olive oil twice a week for 4 weeks	Liver regeneration induced by the *in situ* transformation of the transplanted hDPSCs, with reduced expression of ACTA2, Col1a1, and liver fibrosis-related genes and proteins: MMP-2, MMP-3, TIMP-1, TIMP-2, and TGF-β	Iwanaka et al., 2020
BM-MSCs with recombinant adeno-associated virus expression vector encoding human HGF genome sequence (rAAV-HGF)	Stem Cell Bank of the Chinese Academy of Sciences (CAS)	SD rats fed with 5% ethanol and subcutaneously injected with 40% CCl4 diluted 1:1 (v/v) in olive oil (0.5 ml/kg) 3 times/week for 9 weeks	Reduction of fibrotic structure related to low expression of α-SMA, collagen I, and vimentin transcripts	Sun et al., 2020
Rat ADSCs incubated with eugenol in olive oil (10 mg/ml)	Adipose tissue of 2-month-old male rats	SD-1 rats i.p. injected with 1 ml/kg of CCl4 diluted in olive oil 1:1 (v/v) twice a week for 6 weeks	Amelioration of liver function, reduction of fibrotic markers (type III collagen, hyaluronic acid, hydroxyproline) and inflammatory cytokines (TNF-α, IL-1β, and IL-6), by decreasing the mRNA levels of type 1 collagen, α-SMA, and TGF-β genes	Fathy et al., 2020

In the CCl_4_-induced CLD model, the administration of murine BM-derived-MSCs reduced fibrosis, ameliorated the hypoxic liver microenvironment, and improved liver function. These beneficial effects were correlated with a modulation of the TGF-β1/SMADs signaling pathway in liver cells. In particular, BM-MSCs reduced TGF-β1 and SMAD3 expression and increased SMAD7 expression (Zhang et al., 2015, 2019). SMAD7 can be regulated by different stimuli, including TGF-β, IFN-γ, and TNF-α. The downregulation of SMAD7 expression is associated with both tissue fibrosis and inflammatory disease; instead, its over-expression antagonizes TGF-β-mediated fibrosis and inflammation (Yan et al., 2009).

Another aspect that characterizes in a distinctive manner liver fibrosis is the induction of collagen deposition, with a consequent complete remodeling of the ECM. The interstitial ECM is mainly composed of structural fibrils of type I and type III collagen and in a minor quantity of type V collagen (Karsdal et al., 2020). The analysis of gene expression profile of ECM in MSC-treated fibrotic animals indicated the reduction of the deposition of collagen in the area of injury. The pro-collagen gene expression significantly decreased when MSCs were administrated in liver fibrosis models generated by the injection of CCl_4_ and of TAA chemical drugs (Jang et al., 2014; Farouk et al., 2018), especially when the transplantation of cells occurred *via* the portal vein rather than by the tail vein (Truong et al., 2016). Besides, IL-6 stimulates the activation of STAT3 and increases collagen mRNA expression in HSCs; the phosphorylation of STAT3 activates the TGF-β cascade through SMAD3 activation (O’Reilly et al., 2014). In this framework, Zhang et al. (2019) demonstrated that BM-MSCs reduced fibrosis by modulating the TGF-β1/SMADs signaling. Yuan et al. (2019) showed that the low amount of collagen deposition was related to low IL-6 mRNA levels and the reduction was evident especially for type III collagen α1. Several studies reported the involvement of IL-17A in liver fibrosis (Zepeda-Morales et al., 2016), since the IL-17 receptor complex IL-17RA/IL-17RC induced in HSC the activation of STAT3, which led to increased collagen mRNA expression. After MSC transplantation, the expression of fibrogenic type I collagen α1 mRNA decreased in liver tissue. Moreover, a gradual reduction of the mRNA expression of IL-17a, IL-17f, and IL-17ra and IL-17rc receptors was observed in the BM-MSC-treated group (Farouk et al., 2018).

The reduction in pro-collagen gene expression often correlates with increased secretion of collagen-degrading MMPs (Rengasamy et al., 2017; Du et al., 2018). In fact, the administration of MSCs induces the reduction of fibrosis by increasing the expression of MMP-9, which degrades the ECM (Tanimoto et al., 2013) and modulates genes involved in matrix remodeling, such as MMP-2 and TIMP-1 (Zhao et al., 2012; Rengasamy et al., 2017; Du et al., 2018; Khalifa et al., 2019; Iwanaka et al., 2020).

Mesenchymal stromal cells can influence the fibrosis development indirectly through the reduction of the hepatic inflammatory state. In fact, MSCs display an immunoregulatory activity by preventing the maturation of immune cells. In particular, they inhibit the proliferation of dendritic, T helper-1, and natural killer cells and induce the activation of M2 macrophages through the production of PGE2, IDO, and NO and secretion of anti-inflammatory ILs, such as IL-10 (Aggarwal and Pittenger, 2005). Anti-inflammatory effects of MSCs in liver tissue were evaluated by measuring the expression levels of pro-inflammatory cytokines in different animal models of CLD, such as HFD, MCDD, CCl_4_ infusion, and TAA administration (Zhao et al., 2012; Nasir et al., 2013; Idriss et al., 2018; Bruno et al., 2019). These studies demonstrated that TNF-α, IL-6, and other pro-inflammatory cytokines [IL-1β, TGF-β1, INF-γ, and monocyte chemoattractant protein-1 (MCP-1)] were downregulated in the liver of treated mice (Zhao et al., 2012; Nasir et al., 2013; Idriss et al., 2018; Bruno et al., 2019; Choi et al., 2019; Kojima et al., 2019). Moreover, treatment with MSCs increased the production of the anti-inflammatory IL-10 that may modulate the expression of α-SMA, collagen I, and TGF-β in target cells both *in vitro* and *in vivo* (Rabani et al., 2010; Idriss et al., 2018; Choi et al., 2019).

Although MSC transplantation has shown beneficial effects in liver fibrosis, several issues must be carefully considered, including the injected cell dose and the timing of treatment. The anti-fibrotic effect of MSCs seems to be dose-dependent: in fact, compared with lower doses, higher cell doses showed a significant reduction in collagen release (Hong et al., 2014). Furthermore, the anti-fibrotic effect of MSCs in the liver is more evident when MSCs are administered in the earlier stages of injury (Zhao et al., 2005), whereas no beneficial effects are observed when MSCs are injected after long-term injury (Popp et al., 2007; Carvalho et al., 2008).

## MSC Engraftment and Differentiation Into Liver Tissue

A few studies support the idea of the engraftment and differentiation of MSCs in the damaged tissue (di Bonzo et al., 2008; Stock et al., 2014; Iwanaka et al., 2020). In a model of intoxication of the liver caused by a sub-lethal dose of acetaminophen (APAP) and treated with human MSCs differentiated into hepatocyte-like cells *in vitro* (hMSC-HCs), the engrafted cells were detected in liver section after long-term transplantation (7 weeks after treatment). In particular, hMSC-HCs were localized in the periportal areas of the liver damaged by APAP, expressing both human albumin and HepPar1, a mitochondrial antigen of hepatocytes, indicating an *in situ* differentiation of hMSC-HCs into hepatic precursors. The engraftment of hMSC-HCs did not cause an increment in the collagen content with respect to untreated APAP animals (Stock et al., 2014). Recently, also in CCl_4_-injured liver, it was reported that the hepatic regeneration is due to *in situ* differentiation of the transplanted hDPSCs. The regenerative effect was correlated with the reduced gene expression of α-SMA, collagen 1, and other fibrosis-related genes and proteins, such as MMP-2, MMP-3, TIMP-1, TIMP-2, and TGF-β (Iwanaka et al., 2020).

In contrast, some authors have reported that, after *in vivo* injection, BM-MSCs can differentiate into myofibroblasts, thus contributing to the progression of liver fibrosis (di Bonzo et al., 2008; Baertschiger et al., 2009). di Bonzo et al. (2008) demonstrated that human BM-MSCs were able not only to engraft around the portal tract of both normal and CCl_4_-injured liver of NOD/SCID mice but also to differentiate into hepatic cells in the *in vivo* acute liver injury model (di Bonzo et al., 2008). In the chronic liver injury model, the percentage of cells expressing the human leukocyte antigen (HLA)-I was significantly higher than in the acute setting; however, a significant number of human cells co-expressed markers of myofibroblast-like cells (α-SMA or glial fibrillary acidic protein) and were located around fibrotic areas, indicating a pro-fibrotic effect of MSCs. These evidences correlated in part with the results obtained by Baertschiger et al. (2009) in a murine model of partial hepatectomy associated with the inhibition of endogenous liver regeneration by retrorsine treatment. Following intrasplenic injection of BM-MSCs, the engraftment of these cells was not achieved in the liver. However, after intrahepatic injection, BM-MSCs permanently engrafted into the liver and might contribute to fibrosis by differentiating into myofibroblasts (Baertschiger et al., 2009). Besides, by comparing the *in vivo* effect of adult BM-MSCs with the effect of pediatric BM-MSCs, no difference in MSC engraftment and no evidence of MSC differentiation into hepatocytes were observed (Baertschiger et al., 2009). Whether the transplanted MSCs have a positive or negative effect on resident liver cell populations (HSCs, immune cells, hepatocytes) is an important aspect that needs to be considered (Kallis and Forbes, 2009). Further studies are required to better understand those mechanisms that could induce MSCs or resident liver cells to produce scar or ECM-degrading substances.

## Anti-Fibrotic and Anti-Inflammatory Effects of MSC Secretome

In addition to direct differentiation of MSCs into liver cells, MSCs exert pro-regenerative and anti-fibrotic effects in liver tissue by inducing the proliferation of resident mature hepatocytes or of progenitor cells through the secretion of paracrine factors. Analysis of gene expression of different sources of MSC revealed that MSCs produce several molecules that can inhibit the activation of HSCs *in vitro*, such as IL-10, VEGF-A, and HGF (Aggarwal and Pittenger, 2005). In particular, HGF promotes hepatic regeneration and exerts anti-fibrotic effects by enhancing hepatocyte proliferation and inhibiting apoptosis (Lee et al., 2018). It has been demonstrated that the stable expression of HGF in BM-MSCs improved cell homing capacity and differentiation into liver cells, thus alleviating CCl_4_-induced liver fibrosis in rats (Sun et al., 2020). Therefore, the release of HGF in the parenchyma of damaged livers could be associated with the ability of MSCs to reverse the progression of liver fibrosis.

The MSC secretome has been proven to be safer and equally effective in liver regeneration (Driscoll and Patel, 2019). In fact, the secretome obtained by UC-MSCs, either undifferentiated or committed into hepatocyte-like cells, improved hepatic fibrosis both *in vivo* and *in vitro*. In particular, the UC-MSC secretome was enriched in the milk factor globule EGF 8 (MFGE8), an anti-fibrotic protein, which expression is reduced in fibrotic or cirrhotic livers. The MFGE8-containing secretome reduced the ECM deposition and suppressed the activation of HSCs by downregulating α-SMA expression and the TGF-β signaling pathway (An et al., 2017). A similar anti-fibrotic effect was also observed *in vitro* on TGF-β-activated HSCs, using conditioned medium derived from AMSCs (Fu et al., 2018) and from BM-MSCs (Huang et al., 2016). Interestingly, in a CCl_4_-induced fibrosis murine model, the BM-MSC secretome also showed immunosuppressive properties, by reducing inflammatory infiltration, and pro-regenerative effects, by enhancing hepatocyte proliferation and promoting HSC apoptosis (Huang et al., 2016). Taken together, these findings suggest that the anti-fibrogenic effect of MSCs can be mediated through the release of paracrine factors, which include soluble factors and EVs.

Extracellular vesicles are small membrane particles delimited by a lipid bilayer membrane that are secreted by virtually all cells into the extracellular microenvironment and can be isolated from all biological fluids. EVs display great heterogeneity in size and molecular cargo, and since specific markers to distinguish one vesicle subpopulation from another are still lacking, EVs are currently classified based on their size and biogenesis in exosomes, ectosomes, and apoptotic bodies (Meldolesi, 2018; Bruno et al., 2020a).

Exosomes (30–120 nm) are small EVs that arise from the inward invagination of the membrane of endosomal structures, known as MVBs. Exosomes are released into the extracellular space upon fusion between MVBs and the plasma membrane. This process, called exocytosis, is principally coordinated by the ESCRT machinery (Meldolesi, 2018) and by other ESCRT-associated proteins, such as ALIX that contributes to cargo packaging into vesicles and triggers exosome formation (Baietti et al., 2012; Hurley and Odorizzi, 2012). Besides, an ESCRT-independent mechanism has been described in exosome formation and secretion (Stuffers et al., 2009). Among the proteins participating in this process, the tetraspanins CD63, CD81, and CD9 coordinate the specific sorting of cargo into exosomes (Chairoungdua et al., 2010; Nazarenko et al., 2010; van Niel et al., 2011; Perez-Hernandez et al., 2013), whereas the Rab GTPases Rab11, 27a, 27b, and 35 are involved in vesicle budding, transport along the cytoskeleton, and fusion with the plasma membrane (Savina et al., 2005; Stenmark, 2009; Hsu et al., 2010; Ostrowski et al., 2010; Bobrie et al., 2012; Zhen and Stenmark, 2015). Furthermore, exosome release can be controlled by calcium signaling (Savina et al., 2003), cytoskeleton rearrangements (Granger et al., 2014), and ceramide synthesis (Trajkovic et al., 2008).

Extracellular vesicles generated by the direct outward budding of the plasma membrane are defined as ectosomes or microvesicles (100–1,000 nm). The modifications in the plasma membrane curvature result from changes in protein interactions, which involve the ARRDC1 and the late endosomal protein TSG101 (Nabhan et al., 2012), and from the calcium-dependent activation of enzymes, such as flippases, floppases, scramblases, and calpain, which alter the lipidic composition of the plasma membrane (van Niel et al., 2018). As for exosomes, the process of ectosomes vesiculation and release also relies on cytoskeleton rearrangements, controlled by the ROCK (Li et al., 2012) and the signaling cascade of Ras-related GTPase ARF6 (Muralidharan-Chari et al., 2009). Lastly, fragments of dead cells that are released through the plasma membrane blebbing of apoptotic cells are defined as apoptotic bodies (1,000–5,000 nm) (Hristov et al., 2004).

At present, EVs are considered as important mediators of intercellular communication (Lee et al., 2011; Lawson et al., 2017). In fact, the EV cargo includes proteins, lipids, and nucleic acids that can be transferred between cells protected from degradation, thus eliciting their intracellular uptake by endocytosis. The specific molecular content of EVs usually depends on the tissue of origin, but it can also be genetically modified to allow EVs to carry the desired therapeutic molecules (Varderidou-Minasian and Lorenowicz, 2020). Once internalized by target cells, EVs can modulate a number of physiological and pathophysiological processes, including metabolism, immune responses, tumor progression, and metastasis (Lou et al., 2017a).

## MSC-EV Effects in Liver Fibrosis

**FIGURE 1:**
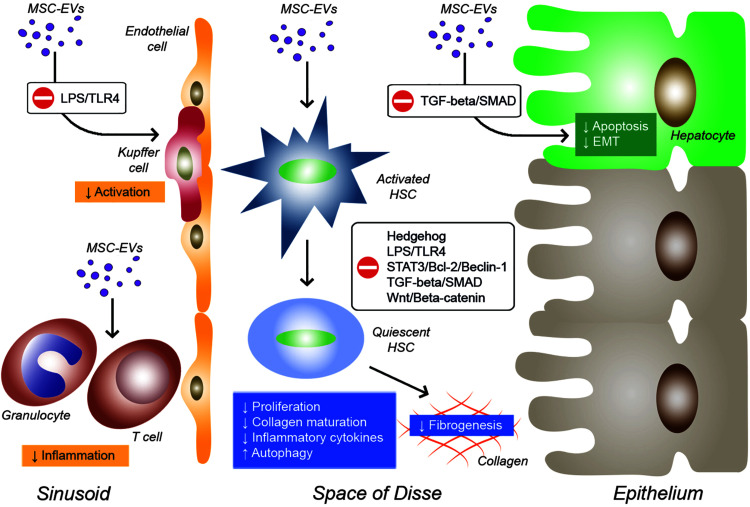
Effects of EVs derived from MSCs on inflammatory cells, Kupffer cells, hepatic stellate cells (HSCs), and hepatocytes during liver fibrosis. EVs from MSCs derived from different sources can modulate various molecular pathways in target cells by reducing inflammation, Kupffer cell activation, HSC activation and fibrogenesis, and hepatocyte apoptosis and EMT.

Recently, the role of EVs in tissue repair and regeneration has been extensively studied. In particular, MSC-derived EVs have shown a pro-regenerative effect in several tissues, including heart, lung, bone, skin, brain, kidney, and liver (Fiore et al., 2018; Varderidou-Minasian and Lorenowicz, 2020). As reported for MSCs, also MSC-EVs exhibit therapeutic effects in several preclinical models of hepatic fibrosis (Table 2). In a context of liver fibrosis, MSC-EVs act by modulating different molecular pathways into hepatocytes, activated HSCs and immune cells (Figure 1).

**TABLE 2:** Anti-fibrotic effect of MSC-EVs in different models of chronic liver injury.

**Source**	**Isolation method**	**Liver fibrosis model**	**Mechanism of action**	**References**
Human UC-MSCs	Differential U.C. Concentration (100 kDa) Density gradient U.C. Filtration (0.22 μm)	CCl4 mouse model. HL7702 human epithelioid liver cell line activated by TGF-β1	Downregulation of fibrotic genes (COL1 and COL3), inactivation of the TGF-β/SMAD pathway (TGF-β1, SMAD2) and of EMT in hepatocytes (E-cadherin, N-cadherin, vimentin)	Li et al., 2013
Human UC-MSCs	Filtration (0.1 μm). Concentration (100 kDa) Density gradient U.C. Filtration (0.22 μm)	Schistosomiasis mouse model. LX2 activated by TGF-β1	Increased mice survival, improvement of liver function by downregulating pro-fibrotic genes (COL1, COL3, and α-SMA) and pro-inflammatory cytokines (TNF-α, IL-1β, and IFN-gamma)	Dong et al., 2020
Human UC-PVCs engineered to produce IGF-1	Differential U.C.	TAA mouse model	Downregulation of pro-fibrotic genes (COL1A2, α-SMA, and TGF-β)	Fiore et al., 2020
Human AMSCs	Differential U.C. Filtration (0.22 μm)	CCl4 rat model. NASH (HFD) rat model. Rat HSC and Kupffer cells activated by LPS	Reduction of Kupffer cell number and HSC activation, by downregulating pro-inflammatory cytokines (TNF-α, IL-1β, and IL-6) and pro-fibrotic genes (TGF-β1, α-SMA, and TIMP-1) and by inactivating the LPS/TLR4 signaling pathway (p65 and IkB-α phosphorylation) in HSCs and in Kupffer cells	Ohara et al., 2018
Human CP-MSCs	Differential U.C.	CCl4 mouse model. FBS-activated LX-2	Reduction in the expression of miR-125b target gene Smo resulted in the downregulation of the Gli family (downstream signaling molecules of Smo) and of the hedgehog signaling pathway in HSC	Hyun et al., 2015
Human ESC-MSCs	Differential U.C.	TAA rat model. Primary hepatocytes	Reduction of fibrosis and immune cell infiltration by upregulating collagenases (MMP-9 and MMP-13) and anti-inflammatory (TGF-β and IL-10) and anti-apoptotic (BCL-2) genes and by downregulating pro-fibrotic (COL1-α, α-SMA, and TIMP-1), pro-apoptotic (caspase-3, BAX), and pro-inflammatory genes (TNF-α and IL-2)	Mardpour et al., 2018
Human ESC-MSCs	Differential U.C.	TAA rat model	Reduction of necrosis, inflammation, and fibrosis by upregulating collagenases (MMP-9 and MMP-13) and anti-inflammatory (IL-10) genes and by downregulating pro-fibrotic (COL1-α, α-SMA, and TIMP-1), pro-apoptotic (cleaved caspase-3, BAX), and pro-inflammatory genes (TNF-α and IL-2)	Mardpour et al., 2019
miR-122-modified murine ADSCs	ExoQuick-TC kit	*In vitro* culture-induced LX-2 activation	Reduction of HSC proliferation and inhibition of the expression of COL1A1 and of miR-122 target genes: CCNG1, IGF1R, and P4HA1	Lou et al., 2017b
miR-181-5p-modified murine BM-MSCs	ExoQuick-TC kit	CCl4 mouse model. HST-T6 cells activated by TGF-β1	Amelioration of liver function, activation of autophagy by upregulating P62 and Beclin1, attenuation of fibrosis and inflammation by downregulating fibrotic genes COL1, COL3 vimentin, α-SMA fibronectin, of miR-181-5p targets Stat3 and Bcl-2, and pro-inflammatory cytokines (TNF-α, IL-6, and IL-17)	Qu et al., 2017
Murine BM-MSCs engineered with miR-223	Differential U.C.	Autoimmune hepatitis mouse model. Murine hepatocytes (AML12) treated with LPS and ATP	Improvement of liver structure and function, reduction of lymphocyte infiltration, with downregulation of pro-inflammatory cytokines (TNF-α, IL-1β, and IL-17A) and of pro-apoptotic proteins NLRP3 and caspase-1	Chen et al., 2018
Human BM-MSCs	Differential U.C.	CCl4 rat model. Activated HSCs	Improvement of liver function and reduction of fibrosis, inflammation and HSC activation through the inhibition of fibrosis-related proteins (COL1, α-SMA) and of the Wnt/β-catenin pathway (PPAR-gamma, β-catenin, Wnt3a, Wnt10b, WISP1, cyclin D1)	Rong et al., 2019
HLSCs	Differential U.C. Filtration (0.22 μm)	NASH (MCDD) mouse model	Improvement of liver function and reduction of fibrosis and inflammation by increasing the anti-inflammatory cytokine IL-10 and by downregulating fibrosis-associated genes (α-SMA, COL1-α1, TGF-β1, and Ltbp1), genes involved in tissue remodeling and in inflammation, such as TIMP-1; MMP-1a, -13, -14, and -8; IFN-γ; TNF-α; and IL-1β	Bruno et al., 2020b

The first evidence that MSC-EVs were able to alleviate liver fibrosis came from Li et al. (2013). In a mouse model of CCl_4_-induced liver fibrosis, they showed that EVs derived from UC-MSCs ameliorated hepatic inflammation and collagen deposition. Moreover, *in vitro* UC-MSC-EV administration suppressed both the EMT and the TGF-β/SMAD signaling pathway through the inhibition of SMAD2 phosphorylation and the reduction of type I and III collagen and TGF-β transcripts expression in hepatocytes. The therapeutic effect of UC-MSC-EVs has been also demonstrated in schistosomiasis, a parasitic disease that leads to serious chronic liver inflammation (Dong et al., 2020). *In vivo* administration of UC-MSC-EVs in *Schistosoma japonicum*-infected mice alleviated hepatic fibrosis by downregulating the expression of α-SMA and types I and III collagen. The consequent inhibition of HSC activation was also confirmed *in vitro* on HSCs. In addition, UC-MSC-EVs reduced the mRNA expression of pro-inflammatory cytokines TNF-α, IL-1β, and IFN-gamma in schistosome-infected liver tissue. Also, EVs obtained from UC-PVCs can effectively reduce both fibrosis and inflammation in a TAA-induced model of chronic liver injury (Fiore et al., 2020). Interestingly, EVs derived from UC-PVCs that were transduced by an adenovirus vector to produce human IGF-1 exhibited a stronger anti-fibrotic effect with respect to their green fluorescent protein (GFP)-transfected counterpart. *In vitro* experiments demonstrated that treatment with IGF-1-containing UC-PVC-EVs reduced the activation of HSCs by downregulating the expression of type I collagen, α-SMA, and TGF-β1. Furthermore, IGF-1-containing UC-PVC-EVs converted pro-fibrogenic hepatic macrophages into anti-inflammatory phagocytes by increasing arginase-1 and downregulating iNOS, TNF-α, and IL-6 expression levels.

Another embryonic-derived source of MSCs is the amnion. In two different models of CLD, Ohara et al. (2018) have demonstrated the anti-inflammatory and anti-fibrotic effects of EVs obtained from AMSCs. In a rat model of NASH, induced by a HFD, AMSC-EVs downregulated the expression of pro-inflammatory cytokines TNF-α, IL-1β, and IL-6 and reduced the activation of pro-inflammatory M1 macrophages (Kupffer cells) in liver tissue. In CCl_4_-induced hepatic fibrosis, treatment with AMSCs attenuated fibrosis by reducing the expression of α-SMA and the number of Kupffer cells. *In vitro*, AMSCs treatment reduced the expression of TNF-α in both HSCs and Kupffer cells activated by LPS and reduced the NF-kB transcriptional activity induced by LPS, through the inhibition of the phosphorylation of IkB-α and p65. Since AMSC-EVs did not affect NF-kB transcriptional activity induced by TRAF, one could speculate that AMSC-EVs might suppress the earlier steps of the LPS/TLR4 signaling pathway. Interestingly, another research study on a rat model of CCl_4_-induced liver fibrosis demonstrated that EVs released by CP-MSCs were able to transfer miR-125b between MSCs and HSCs. The inhibition of miR-125b targets smo, resulting in the suppression of Hedgehog signaling with consequent amelioration of hepatic fibrosis (Hyun et al., 2015).

Recently, ESCs have been identified as an alternative source of MSCs. In a rat model of TAA-induced chronic liver injury, (Mardpour et al., 2018) reported the hepatoprotective effect of EVs obtained from human ESC-derived MSCs. In TAA animals, the ESC-MSC-EVs improved hepatocyte viability and reduced both apoptosis and the expression of pro-fibrotic molecules, such as collagen, α-SMA, and TIMP-1, while increasing the expression of collagenases, such as matrix metalloproteinase MMP-9 and -13. In addition, ESC-MSC-EVs exhibited immunomodulatory properties by reducing immune cell infiltration and modulating the expression of inflammatory cytokines, with a decrease in TNF-α and IL-2 levels and an increase in TGF-β and IL-10 levels. Interestingly, the anti-inflammatory effect of the ESC-MSC-EVs resulted to be stronger than the one of other somatic tissue-derived MSC secretome, such as BM-MSCs and ADSCs (Mardpour et al., 2018). However, the efficiency of EV treatments might be reduced by a rapid clearance of EVs from the target organ. To extend the bioavailability of the EVs in the liver, the use of ESC-MSC-EVs encapsulated in polyethylene glycol macromeres has been proven to be effective in a TAA rat model of hepatic fibrosis. Once injected into the peritoneum cavity, the hydrogel-released EVs were gradually swollen upon biodegradation and progressively released over 1 month, resulting in liver accumulation. Histological and molecular analysis have pointed out that, compared with freely injected EVs, the hydrogel-released EVs had stronger anti-fibrotic, anti-apoptotic, and anti-inflammatory effects in fibrotic liver tissue (Mathieu et al., 2019).

Adipose tissue-derived MSC have also shown therapeutic effects in hepatic fibrosis (Lou et al., 2017b; Qu et al., 2017). Qu et al. (2017) demonstrated that the transient over-expression of miR-181-5p in ADSCs increased their therapeutic potential in a CCl_4_-induced fibrosis model. In addition, the miR-181-5p over-expressing ADSC-EVs induced autophagy in HSCs by inhibiting the STAT3/Bcl-2/Beclin pathway and suppressed HSC activation induced by TGF-β through the reduction of fibrosis-related genes, such as fibronectin and type I and III collagen (Qu et al., 2017). Another study showed that the engineering of ADSCs with a miR-122 over-expressing lentiviral vector made the ADSC-EVs more effective against liver fibrosis than their non-transfected counterpart. In fact, ADSC-EVs efficiently transfer miR-122 to HSCs, inhibiting cell cycle progression and suppressing miR-122-target genes CCNG1, IGF1R, and P4HA1, which regulate proliferation and collagen maturation in HSCs (Lou et al., 2017b).

Extracellular vesicle engineering has been proven effective against liver fibrosis also for MSC-EVs derived from other sources, such as the bone marrow. In a murine model of autoimmune hepatitis, EVs derived from BM-MSCs improved liver function by downregulating the expression of inflammasome and apoptosis-related genes NLRP3 and caspase-1. The lentivirus-driven upregulation of miR-223 in BM-MSCs improved the anti-inflammatory and cytoprotective properties of BM-MSC-EVs, both *in vitro* and *in vivo*, whereas the specific inhibition of miR-223 completely abrogated the therapeutic effect of BM-MSC-EVs (Chen et al., 2018). Besides, the administration of BM-MSC-EVs in CCl_4_-induced liver fibrosis suppressed the Wnt signaling pathway in activated HSCs by downregulating the expression of β-catenin, Wnt3a, Wnt10b, and PPAR-gamma (Rong et al., 2019).

Recently, our group has investigated the therapeutic effect of EVs released by HLSCs in CLD (Bruno et al., 2020b). HLSCs are stem cell populations resident in human adult liver that have MSC-like features (phenotype, gene expression profile, multilineage differentiation, and immunoregulatory capacities). Moreover, HLSCs showed commitment toward hepatic lineage and contributed to tissue regeneration in different experimental animal models of liver injury (Herrera et al., 2006; Bruno et al., 2019). In a murine model of liver fibrosis, HLSC-EVs improved liver function and morphology through the reduction of fibrosis and inflammation. Molecular analyses revealed that HLSC-EV administration downregulated most of the fibrosis-associated genes, such as type I collagen, α-SMA, TGF-β, and the gene latent-TGF-β-binding protein 1 (Ltbp1), which expression was increased by MCDD. The HLSC-EVs treatment also reverted the expression of genes involved in tissue remodeling (MMP-1a, -8, -13, and -14 and TIMP-1) and in inflammation (IFN-gamma, IL-1β, and TNF-α). Furthermore, the increase of IL-10 expression levels and the reduction of inflammatory infiltrations in the liver confirmed the anti-inflammatory potential of HLSC-EVs. Interestingly, proteomic analyses of HLSC-EV cargo revealed a number of anti-inflammatory proteins that might contribute to the improvement of liver fibrosis.

## Conclusion

Mesenchymal stromal cells treatment can reduce fibrosis by downregulating gene expression levels of different transcripts fundamental for fibrosis development, such as α-SMA, TGF-β, and collagens. In liver, the regression of fibrosis could be also attributed to the enhanced levels of MMPs secreted by MSCs that degrade and remodel the fibrotic matrix. Immunomodulatory capacities of MSCs could also be implicated in anti-fibrotic effects by coordinating the recruitment and the polarization of inflammatory cells and the production of cytokines, thus regulating the pro-fibrotic environment. However, it is still debated whether MSCs could engraft into the liver and whether, once engrafted, MSCs could differentiate into hepatocytes, thus participating in liver regeneration, or into pro-fibrotic cells, thus getting worse both liver function and morphology. To avoid the differentiation of MSCs into pro-fibrotic cells, the use of the MSC secretome would be preferable over the MSC treatment. Several research articles indicated that secretome could mimic the effects of cell injection and exert similar anti-fibrotic effects. Among the paracrine factors produced by MSCs, EVs emerge as a valid and alternative tool to cell treatment. Compared with cell-based approach, the EV treatment shows some benefits, such as the possibility of avoiding cell misdifferentiation and the consequent aggravation of liver fibrosis. In fact, to our knowledge, no evidence of fibrogenic potential of MSC-EVs has been reported. In addition, the use of MSC-EVs has a higher efficacy profile than MSC therapy since, once injected *in vivo*, the EVs pass through the biological barriers and shuttle different molecules (proteins, RNAs, and lipids) to target cells and tissues. Interestingly, in the context of CLD, MSC-EVs have been proven to regulate different molecular pathways in liver cells, such as the TGF-β/SMAD, the LPS/TLR4, the STAT3/Bcl-2/Beclin, and the Wnt signaling pathway.

Despite the promising results obtained in CLD preclinical studies with MSC-EVs, many problems need to be solved prior to clinical EV applications. Currently, EV purification is a challenging topic: several protocols have been proposed for EV isolation, and most of them can be combined to achieve higher purity of the EV sample by removing cell debris, protein aggregates, and vesicles of non-endosomal origin, such as lipoproteins (Mathieu et al., 2019). Differential ultracentrifugation represents the “gold standard” for EV purification (Théry et al., 2006), but long times, possible EV damage and contamination by non-EV particles hamper the clinical application of this technology. An alternative approach to isolate distinct EV subtypes is density gradient centrifugation, which consists in an iodixanol gradient able to separate different EV populations based on their density (Iwai et al., 2016). Moreover, the density gradient ultracentrifugation may be combined with floating to separate vesicles from contaminant proteins (Kowal et al., 2016). Immunoaffinity capture technology takes advantage of EV-associated molecules, such as tetraspanins and ESCRT proteins, but the lack of specific EV subpopulation markers makes it difficult to distinguish one type of vesicle from another (Lozano-Andrés et al., 2019). The majority of these techniques are suitable for research but limited to application for scalable production of EVs for therapeutic purposes. Membrane filtration is potentially applicable to large volumes of conditioned medium. This technique has been proven to be effective in separating EV subpopulations based on their density and hydrodynamic properties (Heinemann et al., 2014). Tangential flow filtration has been also proposed for scalable production of EVs from large volumes of conditioned medium (Busatto et al., 2018). Similarly, size exclusion chromatography has revealed one of the most rapid EV isolation techniques, particularly indicated for exosome purification (Blans et al., 2017). Even if EVs appeared more stable and resistant for long-term storage than MSCs, further studies on pharmacological and pharmacodynamic properties are needed to clarify whether the use of MSC-EVs is suitable for clinical application.

## Author Contributions

SB, GCh, and CP performed the research of the pertinent literature and designed and drafted the manuscript. GCa revised and edited the manuscript. All authors contributed to the article and approved the submitted version.

## Conflict of Interest

GCa is a component of Scientific Advisory Board of Unicyte AG. The remaining authors declare that the research was conducted in the absence of any commercial or financial relationships that could be construed as a potential conflict of interest.

## Abbreviations

CLD: chronic liver disease
ECM: extracellular matrix
DAMPs: damage-associated molecular patterns
HSC: hepatic stellate cell
α-SMA: alpha-smooth muscle actin
EMT: epithelial-to-mesenchymal transition
PDGF: platelet-derived growth factor
TGF- β: transforming growth factor beta
TNF- α: tumor necrosis factor alpha
IL: interleukin
CCL2: chemokine (C–C motif) ligand 2
STAT: signal transducer and activator of transcription
PI3K: phosphatidylinositol 3-kinase
MMP: metalloproteinase
TIMP: tissue inhibitor of metalloproteinase
SMAD: small mother against decapentaplegic
NASH: non-alcoholic steatohepatitis
MSC: mesenchymal stromal cell
EV: extracellular vesicle
HpSCs: hepatic stem/progenitor cells
CCl_4_: carbon tetrachloride
BM-MSC: bone marrow-derived MSC
IFN- γ: interferon gamma
TAA: thioacetamide
PGE2: prostaglandin E2
IDO: indoleamine 2,3-dioxygenase
NO: nitric oxide
HFD: high fat diet
MCDD: methionine- and choline-deficient diet
α-FP: alpha-fetoprotein
CK-18: cytokeratin-18
GFAP: glial fibrillary acidic protein
APAP: acetaminophen
hMSC-HC: human MSC differentiated into hepatocyte-like cell *in vitro*
hDPSC: human deciduous pulp stem cell
VEGF: vascular endothelial growth factor
HGF: hepatocyte growth factor
UC-MSC: umbilical cord-derived MSC
MFGE8: milk factor globule EGF
AMSC: amniotic fluid-derived MSC
MVBs: multivesicular bodies
ESCRT: endosomal sorting complex required for transport
ALIX: apoptosis-linked gene-2 interacting protein X
ARRDC1: arrestin domain-containing protein-1
TSG101: tumor susceptibility gene 101
ROCK: Rho-associated protein kinase
ARF6: ADP-ribosylation factor 6
UC-PVC: umbilical cord-derived perivascular cell
IGF-1: insulin growth factor 1
iNOS: inducible nitric oxide synthase
CP-MSC: chorionic plate-derived MSC
ESC: embryonic stem cell
ADSCs: adipose tissue-derived MSC
CCNG1: cyclin G1
IGF1R: insulin-like growth factor receptor 1
P4HA1: prolyl-4-hydroxylase a1
PPAR: peroxisome proliferator-activated receptor
HLSC: human liver stem cell
Ltbp1: latent-transforming growth factor beta-binding protein 1.
